# 

**Published:** 1883-05

**Authors:** 


### INTERNATIONAL HAHNEMANNIAN ASSOCIATION.

### Officers for 1883.

*President*, C. Pearson, M. D., Washington; *Vice-President*, J. P. Mills, M. D., Chicago; *Secretary*, J. B. Gregg Custis, M. D., Washington; *Corresponding Secretary*, E. W. Berridge, M. D., London; *Treasurer*, Edward Cranch, M. D., Erie, Pa.

Board of Censors: Benjamin Ehrman, M. D., Cincinnati; S. Swan, M. D., New York; T. P. Wilson, M. D., Ann Arbor; C. H. Lawton, M. D., Wilmington, Del.; T. F. Pomeroy, M. D., *Chairman*.

### Bureaus.

Bureau of Clinical Medicine: Edward Bayard, M. D., New York; Constantine Lippe, M. D., New York; C. Carleton Smith, M. D., Philadelphia; G. Pompili, M. D., Rome, Italy; C. F. Nichols, M. D., Boston; E. A. Ballard, M. D., Chicago; Rollin R Gregg,* M. D., *Chairman*, Buffalo.

---

* Dr. Gregg requests that all who may have interesting or unusual clinical cases to report to the International Hahnemannian Association, will please send them to him.

BUREAU OF OBSTETRICS AND DISEASES OF WOMEN AND CHILDREN: W. H. Leonard, M. D., Minneapolis, Minn., Some of the Causes of Cancer of the Uterus; John Hall, M. D., Toronto, Canada, Membranous Dysmenorrhœa; Edward Mahoney, M. D., Liverpool, England, Illustrative Cases; George F. Foot, M. D., Stamford Conn., Diseases of Children; Edward G. Rushmore, M. D., Plainfield, N. J., Clinical Cases; Adolph Fellger, M. D., Philadelphia, Pa.; L. A. Rendell, M. D., New Haven, Conn.; J. R. Haynes, M. D., Indianapolis, *Chairman*, Retroverted and Adhered Uterus with Treatment.

BUREAU OF SURGERY: James B. Bell, M. D.; L. M. Kenyon, M. D.; J. A. Beigler, M. D.; T. P. Wilson, M. D.; Edward Cranch, M. D.; L. B. Wells, M. D.; Julius Schmitt, M. D.; H. I. Ostrom, M. D., New York, *Chairman*. Subject for discussion: The Therapeutics of Bone Diseases.

BUREAU OF MATERIA MEDICA: Adolph Lippe, M. D., Our Materia Medica: how to Preserve and Augment it; P. P. Wells, M. D.; T. F. Pomeroy, M. D.; E. W. Berridge, M. D., Proving of Ipecacuanha; Edw. Cranch, M. D.; J. P. Mills, M. D.; O. P. Baer, M. D., *Chairman*, Clinique of Verbascum thaps.

NOTICE.

***All articles for publication, books for review, business communications, etc., must be sent to Editor, 2109 Chestnut Street, Philadelphia.***

PHILADELPHIA:

